# Molecular Basis for the Calcium-Dependent Activation of the Ribonuclease EndoU

**DOI:** 10.21203/rs.3.rs-4654759/v1

**Published:** 2024-07-15

**Authors:** Florian Malard, Kristen Dias, Margaux Baudy, Stéphane Thore, Brune Vialet, Philippe Barthélémy, Sébastien Fribourg, Fedor V Karginov, Sébastien Campagne

**Affiliations:** 1 Univ. Bordeaux, CNRS, INSERM, ARNA, UMR 5320, U1212, F-33000 Bordeaux, France; 2 Univ. Bordeaux, CNRS, INSERM, IECB, US1, UAR 3033, F-33600 Pessac, France; 3 Department of Molecular, Cell and Systems Biology, Institute for Integrative Genome Biology, University of California at Riverside, Riverside, CA, 92521, USA

**Keywords:** EndoU, calcium, RNA, allostery, ribonuclease

## Abstract

Ribonucleases (RNases) are ubiquitous enzymes that process or degrade RNA, essential for cellular functions and immune responses. The EndoU-like superfamily includes endoribonucleases conserved across bacteria, eukaryotes, and certain viruses, with an ancient evolutionary link to the ribonuclease A-like superfamily. Both bacterial EndoU and animal RNase A share a similar fold and function independently of cofactors. In contrast, the eukaryotic EndoU catalytic domain requires divalent metal ions for catalysis, possibly due to an N-terminal extension near the catalytic core. In this study, we used biophysical and computational techniques along with *in vitro* assays to investigate the calcium-dependent activation of human EndoU. We determined the crystal structure of EndoU bound to calcium and found that calcium binding remote from the catalytic triad triggers water-mediated intramolecular signaling and structural changes, activating the enzyme through allostery. Calcium-binding involves residues from both the catalytic core and the N-terminal extension, indicating that the N-terminal extension interacts with the catalytic core to modulate activity in response to calcium. Our findings suggest that similar mechanisms may be present across all eukaryotic EndoUs, highlighting a unique evolutionary adaptation that connects endoribonuclease activity to cellular signaling in eukaryotes.

## Graphical Abstract

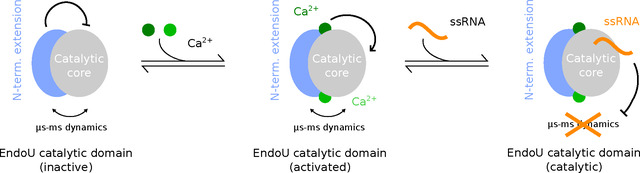

Calcium binds at the interface between the catalytic core and N-terminal extension in eukaryotic EndoU catalytic domains, activating the catalytic site at a distance *via* allostery.

## Introduction

Ribonucleases (RNases) are nucleases that catalyze the processing or degradation of RNA. Found in all organisms, RNases play vital roles in various cellular processes, including maturing both coding and non-coding RNAs, combating RNA viruses, and contributing to sophisticated immune strategies like RNA interference [1, 2, 3]. For example, RNases catalyze mRNA decay in general pathways (XRN1, exosome/DIS3L) or as part of apoptotic cascades (RNase L, DIS3L2), carry out unconventional splicing or tRNA cleavage during stress (IRE1, angiogenin), or catabolize extracellular RNAs (RNase A). Among RNases, the cellular roles of those that cleave endonucleolytically have been increasingly recognized [4]. RNases can be constitutively active (RNase A, angiogenin), or stimulated by ligand binding (RNAse L) or cellular signaling events, such as phosphorylation (IRE1). The ribonuclease A-like domain superfamily (IPR036816 [5]) is the most well-known RNase domain, with many pioneering studies in the 20^th^ century [6, 7]: it was the first directly sequenced enzyme [8], the first enzyme for which a catalytic mechanism was proposed based on experimental data [9], and one of the first solved three-dimensional structures [10]. Despite its significant impact in enzyme research, it is important to note that the RNaseA-like domain is only found in vertebrates, raising questions about its deeper evolutionary ancestors or relatives [6].

The endoribonuclease EndoU-like (Endoribonucleases specific for Uridylate) superfamily (IPR037227 [5]) is a poorly understood group of RNases found in bacteria, eukaryotes and viruses. Notably, a structural similarity between a bacterial EndoU-like toxin and vertebrate RNase A was identified [11]. Furthermore, recent studies uncovered an ancient evolutionary link between the Ribonuclease A and EndoU families, suggesting that the animal RNase A gene could have evolved either through significant alteration of an EndoU gene, or by horizontal acquisition of a prokaryotic ribonuclease [6]. XendoU, the founding member of the EndoU-like superfamily (IPR037227 [5]), was initially identified in *Xenopus laevis* oocyte extracts as an enzyme that releases small nucleolar RNAs from introns [12, 13, 14]. *In vitro* studies demonstrated that XendoU is an endonuclease that cleaves single-stranded RNA preferentially at 5’ of uridylates [15]. In eukaryotes, XendoU defines a distinct EndoU family (IPR018998 [5], PF09412 [16]) that lacks sequence homology with other known RNases, and is broadly conserved across *Arabidopsis thaliana*, *Drosophila melanogaster*, *Mus musculus*, *Homo sapiens*, and other species [14, 15]. Human EndoU (hEndoU) was first identified as human placental protein 11 (PP11) due to its prevalence in the placenta [17, 18]. It is also now recognized as a biomarker in various cancers, including squamous cell carcinomas, ovarian adenocarcinomas, non-trophoblastic tumors and breast cancers [19, 20, 21, 22, 23, 24]. In human cells, EndoU has been proposed to be involved in RNA cleavage, ribonucleoprotein particle removal, and endoplasmic reticulum network organization [25, 26]. Across other eukaryotes, EndoU has been implicated in pro-apoptotic processes in mouse B cells, neuron survival in fruit flies, and synaptic remodeling in nematodes [27, 28, 29]. The more distant bacterial EndoU-like ribonucleases are common in microbial warfare as toxins [30].

Members of the EndoU-like superfamily (IPR037227 [5]) exhibit notable differences in their activation requirements. For instance, it is well characterized that EndoU-like bacterial toxins and arteriviral Nsp11 do not need any cofactors for activation, analogous to vertebrate RNase A [11, 31]. In contrast, studies have shown that purified forms of XendoU and coronaviral Nsp15 require millimolar concentrations of Ca^2+^ or Mn^2+^ [15, 25, 32, 33]. The crystal structure of the endoribonuclease XendoU in the absence of divalent metals has been solved [34], suggesting a catalytic site arrangement similar to that of vertebrate RNase A, specifically featuring a catalytic His-His-Lys triad [34]. However, the structural basis for the metal-dependent activation of eukaryotic EndoUs could not be explained by the crystal structure of XendoU, which represents the inactive state of the endonuclease in the absence of a cofactor [34]. Bacterial and metal-independent viral EndoUs share a smaller, C-terminal catalytic domain compared to eukaryotic EndoUs. Because eukaryotic EndoUs contain an N-terminal extension within this catalytic domain that correlates with Ca^2+^ dependence, we hypothesized that it may bind calcium and control the activity of the catalytic core through allostery.

In this study, we elucidated the molecular mechanism of EndoU activation by calcium. First, we established a thymocyte cell line model to confirm the dependence of EndoU for calcium in both cell extract and recombinant forms. Next, we used biophysical methods to detect an allosteric change upon activation by calcium and to solve the structure of active EndoU. Our structural analysis revealed a calcium-stabilized interaction network involving residues from both the eukaryote-specific N-terminal extension and the catalytic core of EndoU, ultimately leading to the activation of the catalytic triad. Our findings provide unprecedented atomic-level insights into a metal ion-activated member of the EndoU-like superfamily (IPR037227 [5]), addressing a longstanding question in the study of eukaryotic EndoUs, which are of significant interest due to their switchable endonuclease activity.

## Materials and Methods

### Cell culture

VL3–3M2 mouse thymic lymphoma cells [35] were cultured in RPMI 1640 (Corning) supplemented with 10 mM HEPES, 50 μL β-mercaptoethanol, 1 x penicillin/streptomycin, and 10 % fetal bovine serum (FBS). The Platinum-E (Plat-E) retroviral packaging cell line was cultured in DMEM (Corning) supplemented with 10 % FBS (Corning) and 10 units.ml^−1^ of penicillin/streptomycin (Gibco). All cells were grown at 37 °C in an atmosphere containing 5 % CO_2_.

### VL3–3M2 TCR activation

Cell culture 6-well plates were pre-incubated overnight at 37 °C with 1 mL of PBS, either with or without 5 μg.mL^−1^ of anti-CD3e/CD28 or anti-CD3/CD4 antibodies. The PBS was then aspirated, and 5*10^5^ cells in 2 mL of media were added. For PMA/ionomycin stimulation, concentrations of 20 ng.mL^−1^ and 500 ng.mL^−1^ were used, respectively. Total RNA was extracted using Trizol 24 hours later, and RT-qPCR measurements were conducted for EndoU, Rag1, and CD5, normalized against a β-actin control. Fold changes were calculated relative to an unstimulated control.

### EndoU knockout cell generation

EndoU KO VL3–3M2 cells were generated as described [36]. sgRNAs designed to target intron 1 and exon 11 of the EndoU locus (Table S1) were cloned into the pSpCas9(BB)/pX330 Cas9-sgRNA expression plasmid (Addgene #42230). A neomycin resistance cassette flanked by two 900 bp homology regions to intron 1 and exon 11 were assembled into the pUC-19 vector as previously described [36]. The Cas9-sgRNA expression plasmids and the homology arm vector were electroporated into 10^7^ VL3–3M2 cells at 340 V for 47 ms in Opti-MEM (Gibco). Neomycin selection was applied after two days. Clonal cells were subsequently generated and screened via PCR using genomic DNA as the template. This involved primers (Table S1) to detect genomic DNA (positive control for WT and KO, gDNA F/R, 800 bp amplicon), primers to verify the presence of the WT allele (EndoU validation F/R, 1079 bp amplicon), and primers to identify the KO allele (EndoU validation F/Resistance R, 999 bp amplicon).

### Mouse EndoU tagged and mutant constructs

The mouse EndoU cDNA (NM_001168693) was PCR amplified from VL3–3M2 cDNA with primers containing XhoI (forward) and BglII (reverse) restriction sites and ligated into the pMSCV-PIG or pRL-TK vectors. The Q5 site-directed mutagenesis kit (NEB Cat. E0554S) was used to add a C-terminal FLAG-HA tag, or to create the E285A;H286A catalytically dead mutant version, in pMSCV-PIG.

### Viral production and stable integration of EndoU rescue constructs

VL3–3M2 clonal EndoU knockout cells were rescued through viral integration of the above EndoU constructs. Plat-E cells were calcium-phosphate transfected with 10 μg of pMSCV-PIG and 2.5 μg VSVG to produce amphitropic VSVG-pseudotyped retrovirus.

### RT-qPCR

RNA was extracted from whole cells using ribozol followed by two phenol chloroform (pH 5.2) extractions. Superscript II reverse transcriptase was used for cDNA synthesis with 1 μg of total RNA as template. TaqMan probes against EndoU (Cat. 4351372) were used in the RT-qPCR.

### Cell lysis

Cell lysis was carried out by first washing the cells once with PBS buffer and then resuspending them in hypotonic lysis buffer (10 mM Tris-HCl pH 7.5, 10 mM KCl, 5 mM DTT, protease inhibitor). The cells were subsequently incubated on ice for 20 minutes. Isotonicity was restored by adjusting the KCl concentration to 100 mM using a 5 X supplemental buffer (450 mM KCl, 0.08 U.μl^−1^ RNAseIN). In certain experiments, lysates were centrifuged at 17 000 g for 20 minutes to separate the cytoplasmic fraction and collect the supernatant.

### Immunoprecipitations

Immunoprecipitations were carried out using protein A Dynabeads. Beads were prepared by incubation with 16.7 μg.ml^−1^ anti-mouse Fcγ bridging antibody and 16.7 μg.ml^−1^ mouse anti-HA.11 antibody, sequentially. Cell lysates were incubated with prepared beads for 1 hour at room temperature. To equalize the amount of EndoU across reactions, an excess of cell lysate over bead capacity was used, and saturation of EndoU binding was verified by western blot.

### On-bead mouse EndoU RNase assays

In a total volume of 10 μL, reactions consisted of (unless used as a variable) 2 mM calcium, 100 mM Tris-HCl (pH 7.5), 10 mM NaCl, 5 μg of total cytoplasmic RNA, or 1 μM of specific RNA oligo (Table S2, typically 50 mer 1), and the immunoprecipitated EndoU. Reactions were incubated at 37 °C, RNA was extracted and run on an 8 % urea-PAGE gel, and visualized by SYBR Green II. Densitometry was used to quantify substrate degradation using Quantity One (BioRad). Experiments were done in triplicate from distinct samples, with central tendencies expressed as means and variations as standard deviation.

### Production of human EndoU

The open reading frame (ORF) encoding the catalytic domain of human EndoU (135–410) was sub-cloned into the pET24b(+) plasmid (Kan^R^) downstream of the GB1 protein ORF followed by a hexahistidine tag and a TEV protease cleavage site. Expression of EndoU was achieved in *Escherichia coli* BL21 Rosetta (DE3) pLysS. The bacteria were grown in rich LB medium or in M9 minimal medium supplemented with ^15^N-labeled NH_4_Cl (1 g.L^−1^) and ^13^C-labeled glucose (2 g.L^−1^) to achieve uniform isotope labeling. The cultures were grown at 37 °C until reaching an OD_600_ of approximately 0.6. Subsequently, protein expression was induced using 0.25 mM IPTG at 15 °C over 16 hours. The bacteria were harvested by centrifugation (5000 g, 10 min, 4 °C), and the resulting pellets were resuspended in ice-cold lysis buffer (20 mM Tris pH 8, 500 mM NaCl, 250 μL.L^−1^ β-mercaptoethanol). This buffer was further supplemented with 1 mg.mL^−1^ lysozyme and 10 μL.L^−1^ DNase (NEB). Cell lysis was achieved by sonication, running three cycles of 5 minutes each at 20 % amplitude, with 20-second on/off intervals. The lysate was clarified by centrifugation (20000 g, 30 min, 4°C) and the supernatant was loaded onto a gravity-flow histidine affinity chromatography column equilibrated with loading buffer (20 mM Tris pH 8, 500 mM NaCl, 250 μL.L^−1^ β-mercaptoethanol). The column was washed with 15 mM imidazole (10 CV), and the protein was eluted with 300 mM imidazole (5 CV). The eluted protein was then dialyzed against TEV digestion buffer (10 mM Tris pH 8, 250 mM NaCl, 125 μL.L^−1^ β-mercaptoethanol) over 16 hours, in the presence of His_6_-TEV protease (1:10 w/w ratio) to digest the GB1-His_6_ tag. Post-digestion, EndoU was isolated from the flow-through fraction following its loading onto a gravity-flow histidine affinity chromatography column, and washing with the loading buffer (5 CV). The resulting protein was concentrated, and a large excess of EDTA (250 mM) was added to chelate potential divalent cations. Further purification was achieved using a Superdex 75 column pre-equilibrated with storage buffer (10 mM Tris pH 7, 50 mM NaCl, 1 mM TCEP). Finally, EndoU was concentrated to a concentration of 500 μM. It was used immediately for enzymatic assays, while it was stored at −80 °C for other experiments. Point mutants were generated using the QuickChange protocol [37] and EndoU variants were purified using the same protocol as the wild type protein. The sequences of the oligonucleotides are given (Table S3).

### Oligonucleotides synthesis

Oligonucleotides were synthesized using the β-phosphoramidite method with an H8 automated synthesizer (K&A Labs, Germany) on a micromolar scale. For the synthesis of 2’F RNA analogs, sequences started with a Unylinker solid support (Glen Research), and nucleotides were added sequentially using 2’F phosphoramidites. For the synthesis of 3’ labeled Cyanine 5 RNA, the dye was directly attached to the support, and RNA monomers were used. All phosphoramidites and the Cyanine 5 solid support were purchased from LINK (Scotland). Deprotection of the oligonucleotides was performed according to the suppliers protocols. The concentrated crude oligonucleotides were then resuspended in water. The sample concentration was determined from the absorbance at 260 nm and the molar extinction coefficient of the oligonucleotide. This value was calculated using the Integrated DNA Technology online oligo analyzer tool, which uses the standard nearest neighbor method.

### Nuclear Magnetic Resonance

Nuclear Magnetic Resonance (NMR) spectroscopy was used to analyze protein structure and dynamics. Experiments were performed using either a Bruker AVIII NMR spectrometer at 700 MHz with a room-temperature probe, or a Bruker Avance NEO spectrometer at 800 MHz with a cryogenic 5 mm TCI ^1^H-^13^C/^15^N/^2^H Z-gradient probe. These experiments were carried out at 35 °C in a minimal buffer composed of 10 mM Tris (pH 7), 50 mM NaCl, 1 mM TCEP, and 10 % D_2_O for field frequency lock. We acquired 2D ^1^H-^15^N and ^1^H-^13^C correlation spectra using the SOFAST-HMQC experiment scheme [38]. Sequence-specific backbone assignments of ^15^N^13^C-labeled calcium-activated EndoU were achieved via classical 3D triple resonance experiments based on the BEST-TROSY principle [39, 40]. The same approach was applied to EndoU bound to RNA targets. Spectra processing was conducted with Topspin 4 (Bruker) and analyzed using CARA [41] and CCPNMR software 2.4 [42]. Combined ^1^H-^15^N chemical shift perturbations ($\Delta \delta_{\text{comb}}$) were calculated as $\Delta \delta_{comb}=\sqrt{\Delta \delta^{1}H\;+\;0.14\;*\;\Delta \delta^{15}N}$, where $\Delta \delta^{1}H$ and $\Delta \delta^{15}N$ are the chemical shift perturbations (in ppm) for ^1^H and ^15^N resonances, respectively [43]. NMR titrations to map the RNA binding surface on calcium-bound EndoU were performed using a non-cleavable, 2’-fluorinated RNA obtained in house via solid-phase synthesis with the following sequence: 5’-AAGUCC-3’.

### Structure Determination

A sample of the catalytic domain of human EndoU, spanning residues 135 to 410, was prepared at a concentration of 12 mg.mL^−1^ in a buffer containing 10 mM Tris pH 7, 50 mM NaCl, 1 mM TCEP, and 20 mM CaCl_2_. The crystallization of EndoU was carried out at 20 °C using the MCSG4 matrix screen, specifically condition F6, which comprises 0.1 M sodium acetate, 0.1 M HEPES pH 7.5, and 22 % PEG 4k. The resulting crystals were flash-frozen in liquid nitrogen using a cryoprotectant solution identical to the crystallization condition but supplemented with 20 % ethylene glycol. Diffraction data were collected at the SOLEIL synchrotron on the PX1 beamline and processed using XDS [44]. Molecular replacement was conducted with Phaser [45] from the Phenix suite [46], using the AlphaFold 2 [47] predicted structure of the human EndoU protein as the model. This process identified two molecules per asymmetric unit, which were subsequently refined using Phenix and BUSTER [48]. Detailed crystallographic data and refinement statistics are presented in the supplementary materials (Table S4).

### Enzymatic RNA Degradation Assays

RNA degradation assays on the human catalytic domain were carried out to assess the relative activity of EndoU and its variants. An RNA sequence, 5’-CAGGUUUCCCCAACGAAAAAAAAAA-3’, was obtained in-house via solid-phase synthesis. The RNA was labeled at the 3’ end with a Cyanine-5 (Cy5) fluorescent probe for detection purposes. In each assay, EndoU or one of its variants was prepared at a final concentration of 1 nM in presence of 1 μM of the RNA. The enzymatic reaction was initiated by introducing 2 mM CaCl_2_ into the mixture. Samples were collected at 15 time points: 0, 1, 3, 5, 10, 15, 20, 25, 30, 40, 50, 60, 80, 100, and 120 minutes. The reaction was terminated at each time point with an excess of EDTA to chelate calcium ions in order to prevent EndoU activation and further RNA degradation. RNA degradation was monitored by resolving the samples on a polyacrylamide gel containing 6 M urea, followed by electrophoresis at 250 V for 50 minutes. The gel was then scanned with a fluorescence scanner. We used the GelAnalyzer software [49] to integrate band intensities, which were normalized relative to the zero time point. Each assay was conducted in triplicate to ensure the reproducibility of the results. Measurements were taken from distinct samples for each replicate; central tendencies are expressed as means, and variations as standard deviations. Data were processed and analyzed using custom Python scripts. A first-order reaction model, $A\;*\;\exp\left(\pi -k\;*\;t\right)$, was used to fit the enzymatic progress curve, using the *curve_fit* function from the *scipy.optimize* module for regression [50]. The fitted reaction rate *k* characterizes the activity of each EndoU variant. To enable comparison across different variants, this reaction rate was subsequently expressed in relative terms with respect to the wild-type EndoU, yielding a dimensionless parameter.

### Molecular Dynamics

Molecular dynamics simulations were performed using the GROMACS software package (version 2022.1) [51]. System preparation was achieved through CHARMM-GUI and the Input Generator module [52, 53], which was also used to apply single amino-acid substitutions for EndoU variants. To monitor the stability of the calcium binding sites, we used the crystal structure of EndoU bound to calcium ions as input. To monitor the binding of calcium to apo-EndoU, we removed calcium ions from the crystal structure and used the resulting structure as input. Calcium ions were then reintroduced into the system as salt ions. To propose an ensemble of models of calcium-activated EndoU in complex with RNA, we used the AlphaFold (AF) 3 [54] webserver with the human EndoU sequence (135–410), a (U)_6_ RNA, and four calcium ions as inputs. The top-ranked model accurately reproduced each of the calcium binding sites, with an RMSD of 0.313 Å between the crystal structure of calcium-activated EndoU and the corresponding part of the AF3 model. Therefore, we created a hybrid model comprising the experimental structure of calcium-activated EndoU in complex with the AF3-modeled bound RNA. This resulting model was used as input for MD simulations. For all simulations, we used the CHARMM36m force field [55] and the TIP3P water model. Each system was solvated in a cubic box with a 1.0 nm buffer zone between the protein and the box edge, and 50 mM NaCl was added with adjustments to neutralize the system. After downloading the generated inputs, energy minimization was executed in GROMACS using the steepest descent algorithm until the maximum force was below 1000 kJ.mol^−1^.nm^−1^, and then equilibration was done under NVT conditions for 125 ps. Particle Mesh Ewald was used for long-range electrostatics, with a cutoff of 1.0 nm for van der Waals interactions, and a time step of 2 fs was applied. The production phase of the simulations was carried out for 1 μs under NPT conditions at a temperature of 35 °C and a pressure of 1 bar. The output was then analyzed for various parameters using the built-in tools of GROMACS.

### SEC-SAXS experiments

SEC-SAXS experiments were conducted on the SWING beamline at the SOLEIL synchrotron (Saint-Aubin, France). All procedures were carried out at a temperature of 35 °C using a buffer composed of 10 mM Tris pH 7, 50 mM NaCl, and 1 mM TCEP. EndoU was prepared to a concentration of 500 μM. A volume of 75 μL was injected onto a size exclusion column (Bio-SEC 3 Agilent 100 Å), and was then eluted directly into the SAXS flow-through capillary cell at a flow rate of 0.3 mL.min^−1^. SAXS data were collected using an EigerX 4M detector situated 2 m away, using the definition of the momentum transfer $q:q=4\pi \;\sin\left(\theta \right)/\lambda$, where 2θ represents the scattering angle and *λ* the X-ray wavelength (1.033 Å for these experiments). The overall SEC-SAXS setup has been described in earlier publications [56, 57, 58]. A total of 900 SAXS frames were continuously recorded during elution, each with a duration of 1.99 s and a 0.01 s dead time between frames. 180 frames were collected before the dead volume to account for buffer scattering. Data reduction to absolute units, buffer subtraction, and averaging of identical frames corresponding to the elution peak were performed using the in-house SWING software FOXTROT [57] and BioXTAS [59]. BioXTAS was also employed to compute the gyration ratio and to estimate the molecular weight based on the volume of correlation [60]. The fitting of the EndoU homology model to the experimental SAXS data were accomplished through the Crysol software, part of the ATSAS Suite [61, 62].

### Intrinsic Fluorescence

To assess the impact of calcium binding on the tertiary structure of EndoU, we measured the intrinsic fluorescence of the protein with a temperature-controlled spectrofluorometer (FS5, Edinburgh Instruments). Protein samples were prepared at 10 μM in a buffer of 10 mM Tris pH 7, 50 mM NaCl, and 1 mM TCEP, and their fluorescence emission spectra were recorded at 35 °C. Emissions from 300 to 525 nm were recorded to detect fluorescence from tryptophan, tyrosine, and phenylalanine. Slit widths for excitation and emission were set at 5 nm, and each spectrum was an average of three scans, corrected for buffer baseline.

## Results

### EndoU Expression and RNase Activity in a Thymocyte Model

**Figure 1:**
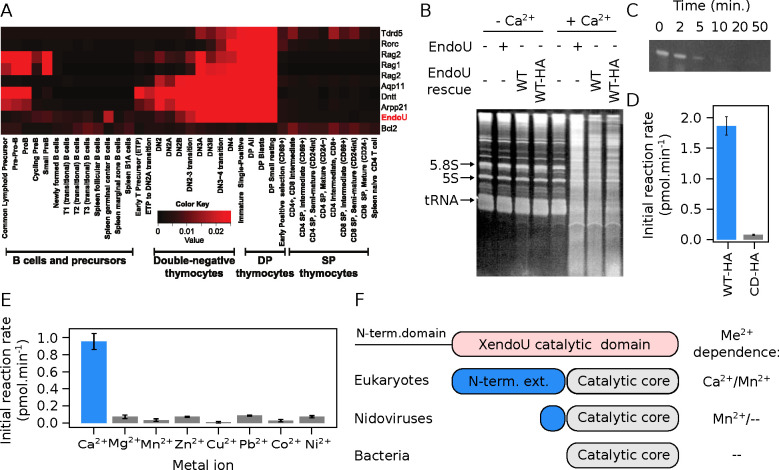
Mouse EndoU is expressed during thymocyte development and has a calcium-activated RNase activity. (A) EndoU expression levels in developing thymocyte populations. The color indicates fractional expression of the mRNA across 211 measured cell types. Data from the Immunological Genome Project [63]. (B) RNase activity in WT and EndoU KO cell lysates. Cytoplasmic lysates from the indicated WT, KO or rescue cell lines were incubated +/− 5 mM Ca^2+^ for 15 minutes at 37 °C. RNA was extracted, 5 μg were run on an 8 % urea-PAGE gel, and visualized by SYBR Green II. WT-HA denotes a WT EndoU rescue construct with a C-terminal HA tag. (C) Immunoprecipitated EndoU cleavage activity on a defined RNA substrate. (D) Mutation of presumed catalytic site residues abolishes EndoU cleavage activity. (E) EndoU RNase activity is specifically activated by Ca^2+^ ions. (F) Domain structure of EndoU and homologs.

In mammals, EndoU expression is limited to specific cell types. Analysis of Immunological Genome Project data [63] on mRNA from 211 mouse hematopoietic cell types revealed strong EndoU expression in developing thymocytes, starting at the double negative (DN) 2–3 transition and progressing through the double positive (DP) stages (Fig. 1 A). EndoU expression is absent in the later stages: single positive thymocytes that survive selection and circulating T cells. Outside the hematopoietic system, EndoU protein staining in human samples [64] showed cytoplasmic expression in stratified squamous epithelia (*e.g.,* skin, esophagus, cervix) and the trophoblast layer in the placenta.

For molecular and biochemical analysis of EndoU, we used the mouse thymic lymphoma cell line VL3–3M2 [35], which resembles double positive thymocytes with high EndoU expression (Fig. S1 A). Upon stimulation with PMA/ionomycin, anti-CD3/CD28, or anti-CD3/CD4, the cell line shows further maturation, including downregulation of Rag1 and EndoU and upregulation of CD5 (Fig. S1 B). EndoU was knocked out in VL3–3M2 cells using CRISPR/Cas9. We confirmed the deletion of the genomic region (Fig. S1 C) and the loss of EndoU mRNA (Fig. S1 D). We assayed endogenous ribonuclease activity in WT and EndoU KO VL3–3M2 extracts, based on experiments in *Xenopus laevis* egg extracts [25]. Incubation of cytoplasmic extracts at 37 °C for 15 minutes without divalent metals caused no RNA degradation (Fig. 1 B). However, WT extracts with 5 mM Ca^2+^ showed robust RNA cleavage, which was absent in EndoU KO extracts and rescued by expressing WT or HA-tagged EndoU in KO cells (Fig. 1 B). Thus, VL3–3M2 extracts have strong EndoU- and Ca^2+^-dependent ribonuclease activity.

To confirm the role and enzymatic properties of EndoU, we used HA-tagged EndoU rescue cells for on-bead *in vitro* cleavage assays with immunoprecipitated EndoU. Since mouse EndoU cleaved various RNA sequences (Fig. S2 A), we used an arbitrary substrate (50 mer 1) for subsequent experiments (Table S2). Time course measurements (Fig. S1 B) were used to calculate initial reaction rates (Fig. S2 C). Mutation of two critical residues [65] abolished activity (Fig. 1 D), confirming the role of EndoU in RNA cleavage. We showed that only Ca^2+^ stimulated cleavage, unlike Mn^2+^ or other divalent metals (Fig. 1 E, S2 B), with optimal activity at 1–2 mM Ca^2+^. EndoU showed little dependence on Na^+^ or K^+^ and sustained activity across pH 4–8 (Fig. S2 D, E, F). These results indicate EndoU is a calcium-activated ribonuclease targeting a large repertoire of RNAs.

### Structural Basis for EndoU Activation by Calcium Ions

To examine structural changes in EndoU upon Ca^2+^ activation, we first computed a homology model of human EndoU using XendoU crystal structure [34] as template via SWISS-MODEL [66]. The apo-EndoU structure is globular, with a predominantly β-sheet catalytic core and an α-helical bundle N-terminal extension (Fig. S3 A). We expressed the XendoU catalytic domain (Fig. 1 D) of human EndoU with ^15^N labeling for NMR spectroscopy. The ^15^N SOFAST-HMQC spectrum of apo-EndoU showed well-dispersed signals but fewer than expected, suggesting conformational exchange in the μs-ms range (Fig. S3 B). Size Exclusion Chromatography with Small-Angle X-ray Scattering (SEC-SAXS) validated the correct folding of recombinant EndoU, matching theoretical predictions (Fig. S3 C). Furthermore, the crysol program [67] showed strong correlation between experimental and theoretical SAXS data, validating the structural model (Fig. S3 D).

We then studied the effect of divalent metal ions on EndoU structure and dynamics by comparing ^15^N SOFAST-HMQC spectra of apo-EndoU to metal-bound states. Saturating concentrations of magnesium, nickel, or strontium caused signal loss in the NMR spectra, suggesting either protein aggregation or increased conformational exchange (Fig. S4 A, B, C). In contrast, saturating calcium restored a set of well-dispersed peaks in the NMR spectrum (Fig. S4 D). At sub-saturating calcium levels, we observed chemical shift perturbations and a shift in the fluorescence spectrum of the protein (Fig. S5 A, B). Intriguingly, the addition of a 2’-fluorinated nonhydrolyzable RNA in the presence of sub-saturating calcium produced effects similar to those observed with saturating calcium, including the restoration of a set of well-dispersed peaks (Fig. S5 C). This was not observed in the absence of calcium, where the substrate analog did not significantly alter the NMR spectrum (Fig. S5 D). These findings suggests a two-step activation process involving local structural changes at lower calcium concentrations and the abrogation of conformational exchange in the μs-ms range at higher calcium concentrations or upon binding of a substrate analog.

**Figure 2:**
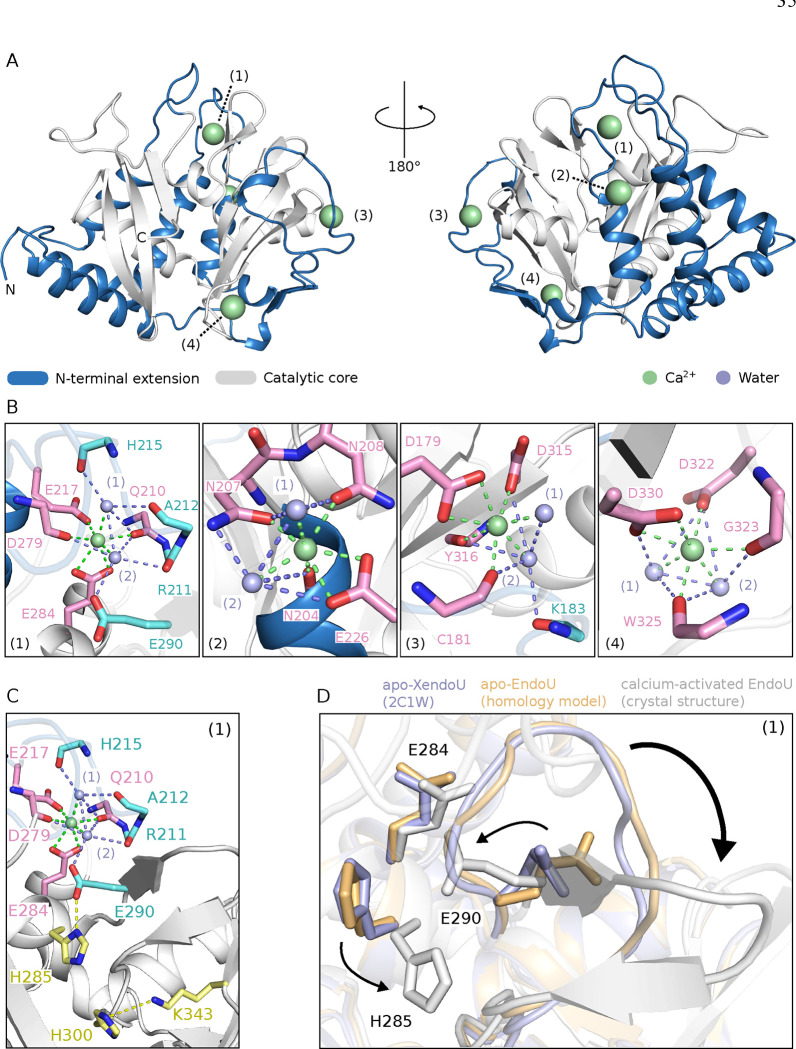
Crystal structure of calcium-activated EndoU. (A) Overview of calcium-activated EndoU structure. Calcium binding site (1) to (4) as refered in the text are indicated. (B) Close-up view of EndoU calcium binding sites. Residues coordinating calcium directly (pink) or indirectly through water-mediated contacts (cyan) are highlighted. (C) Intramolecular signaling between calcium binding site (1) and remote catalytic residues. Catalytic residue (yellow) are highlighted, along with residues coordinating calcium directly (pink) or indirectly (cyan). (D) Structural change upon EndoU allosteric activation by calcium.

To elucidate the effect of calcium, we crystallized EndoU with an excess of calcium and solved its structure at 1.7 Å resolution. The structure revealed five calcium ions, with one aiding crystal packing and four potentially activating the protein (Fig. 2 A, S6). Each calcium ion is coordinated by seven oxygen atoms from acidic side-chains, backbone carbonyl groups, or protein-stabilized water molecules (Fig. 2 B). Sites (1) and (3) include residues from both the catalytic core and the eukaryote-specific N-terminal extension (Fig. S7), with site (1) located 12.8 Å away from the catalytic triad (H285, H300, K343). Comparing apo-XendoU, apo-EndoU, and calcium-activated EndoU structures revealed conformational changes upon calcium binding that mainly cluster nearby protein loops (Fig. 2 C, D). The side-chain of E290, located midway between site (1) and the catalytic triad, flips to engage with a water molecule in the calcium coordination network. This correlates with the side-chain rotation of catalytic H285, then locked by an electrostatic interaction with E290 (Fig. 2 C). The bonding correlates with a disorder-to-order transition in the loop carrying E290, forming a β-hairpin and stabilizing the catalytic site (Fig. 2 D). Our data show a model for the calcium-dependent regulation of EndoU through allostery, with site (1) and residue E290 as key mediators in the intramolecular signaling leading to EndoU activation.

### Experimental Validation of EndoU Activation Model

**Figure 3:**
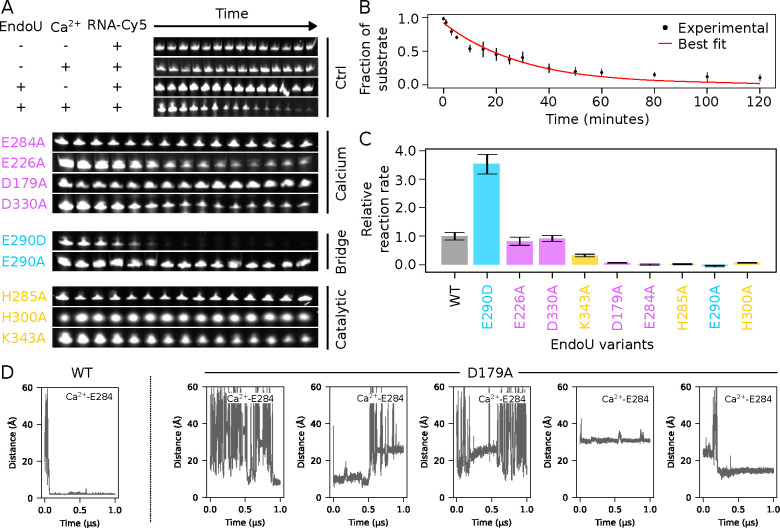
Enzymatic activity of EndoU and its variants. (A) RNA degradation assays. Comparison of mutants for calcium-binding sites (magenta), the bridging residue E290 (cyan), and catalytic residues (yellow) with wild-type EndoU over a 2 hrs degradation assay. (B) Enzymatic progress curve. Example of fit for a first-order reaction model $A\;\times \;\exp\left(-k\;\times \;t\right)$ with wild-type EndoU. (C) Relative reaction rates of EndoU variants compared to wild-type. (D) Calcium binding to EndoU monitored through Molecular Dynamics. Each plot displays the distance between the E284 side-chain carboxylate and a calcium ion throughout the simulation. Five calcium ions were introduced in the simulation box.

To validate our structure-based model for calcium-mediated EndoU activation, we first designed variants with altered calcium-binding sites. RNA degradation assays were conducted for each variant (Fig. 3 A). Without calcium or wild-type EndoU, no RNA cleavage was detected, whereas their presence led to almost complete RNA degradation over time. The degradation data fitted a first-order reaction model, providing a kinetic parameter describing the reaction (Fig. 3 B). Disrupting calcium binding sites (2) or (4) with mutations E226A or D330A led to RNA degradation rates similar to the wild-type (Fig. 3 A, C). In contrast, disruption of calcium binding sites (1) or (3) with mutations E284A or D179A abolished enzymatic activity. Interestingly, calcium binding sites (1) and (3) are defined by residues from both the catalytic core and the eukaryote-specific N-terminal extension, while this is not the case for sites (2) and (4) (Fig. S7).

Our results clearly indicate that the eukaryote-specific N-terminal extension of EndoU contributes to calcium sensing, thereby enabling allosteric regulation. Even though the crystal structure of calcium-activated EndoU could explain the role of calcium binding site (1) in this process, it was not the case for site (3). We hypothesized that calcium binding to site (1) could be promoted by a prior binding event at site (3) and relied on Molecular Dynamics (MD) experiments to test this hypothesis. With the wild-type EndoU, we observed calcium binding to site (1) within less than 100 ns simulation time (Fig. 3 D). Disrupting site (3) with mutation D179A resulted in no stable binding at site (1) for any of the five calcium ions added in the 1 μs simulation. This suggests that that cooperative binding of calcium at sites (1) and (3) leads to EndoU activation.

We further proposed that calcium sensing information at site (1) was communicated to the remote catalytic site through water-mediated intramolecular signaling events enabled by key residue E290 (Fig. 2 C). Disruption of the intramolecular signaling cascade with mutation E290A completely abrogated activity, while charge-conservative mutation E290D resulted in a 4-fold increase in the enzymatic reaction rate (Fig. 3A, C). Consistent with our structural model, a negatively charged side-chain at position 290 is required for the allosteric activation of EndoU by calcium. In this model, E290 locks the catalytic H285 side-chain in an active conformation. Accordingly, substitution of catalytic H285 with alanine completely abrogated enzymatic activity, as observed for catalytic mutant H300A, underscoring the importance of the histidine pair in catalysis. Substitution of catalytic residue K343 by alanine resulted in nearly half reduction of enzymatic activity, consistent with the role of K343 as a stabilizer of reaction intermediates. Overall, mutagenesis experiments corroborate the residue assignments proposed in our structure-based model for calcium-mediated activation of EndoU.

### Calcium-Activated EndoU in Complex with an RNA Analog

**Figure 4:**
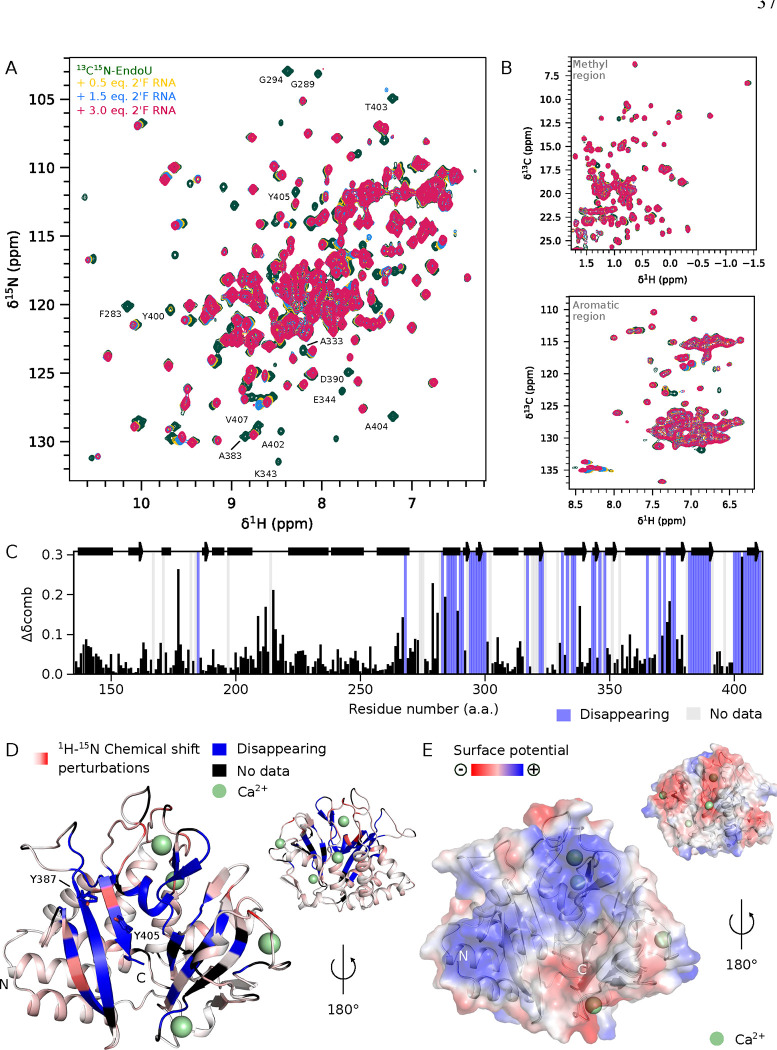
NMR mapping of the RNA binding interface on calcium-activated EndoU. Overlay of (A) ^15^N or (B) ^13^C SOFAST-HMQC spectra from isolated ^13^C^15^N-EndoU (350 μM, green) and upon successive additions of 2’ fluorinated RNA. (C) Combined ^1^H-^15^N chemical shift perturbations between calcium-activated ^13^C^15^N-EndoU and in complex with 2’F RNA. (D) RNA binding interface mapping on calcium-activated EndoU. (E) Electrostatic surface potential of calcium-activated EndoU.

To experimentally determine the RNA-binding surface of calcium-activated EndoU, we first performed 3D triple resonance NMR experiments for backbone chemical shift assignment, successfully assigning 89.3 % (251 out of 281 residues) of the backbone resonances (Fig. S8). Excluding the N-terminal GGSEFA sequence and nine proline residues, the assignment coverage increased to 94.3 %. Next, we recorded ^15^N and ^13^C SOFAST-HMQC spectra of calcium-activated ^13^C^15^N-labeled EndoU with a 2’-fluorinated RNA substrate analog (Fig. 4 A, B). Severe line broadening in a subset of crosspeaks and additional spectral changes in the rapid exchange regime were observed (Fig. 4 A). We calculated ^1^H-^15^N chemical shift perturbations between RNA-bound and unbound EndoU, noting particularly the residues that disappeared in the bound state (Fig. 4 C). All disappearing residues are located in the C-terminal catalytic core, covering the β-sheet surface composed of two independent β-sheets and a short α-helix (Fig. 4 D). The strongest chemical shift perturbations also correspond to residues in this area. Electrostatic analysis indicated that the β-sheet is highly basic, suitable for RNA binding (Fig. 4 E). In contrast, the N-terminal α-helical region showed minimal changes upon RNA binding, with no disappearing resonances, and displayed a neutral or acidic surface charge. These data support that RNA binds to the conserved catalytic core of the protein, involving an extended, basic β-sheet-rich groove for RNA binding.

To model the complex between calcium-activated EndoU and RNA, we used AlphaFold (AF) 3 [54] with the primary sequence of EndoU, a (U)_6_ RNA, and four calcium ions as inputs. We controlled that a 2’F (U)_6_ RNA analog interacts with calcium-activated EndoU (Fig. S9). Using the top-ranked AF model as input, we then conducted a 1 μs Molecular Dynamics (MD) simulation using the top-ranked AF model as input. Over the trajectory, we clustered structures based on RNA conformations to obtain a final ensemble of RNA-bound calcium-activated EndoU models (Fig. S10 A). All models consistently reproduced the crystal structure of calcium-activated EndoU, with a mean RMSD of 1.18 ± 0.29 Å. The intermolecular interface with the (U)_6_ RNA is defined by the cleft between the two front β-sheets, where nucleotide U_2_ anchors in close proximity to the catalytic triad, exposing its sugar 2’OH for nucleophilic attack by residue H300 (Fig. S10 B, S11). Conformational heterogeneity is observed at the interface with EndoU across the models for the rest of the RNA sequence, consistent with the experimental NMR data showing line broadening for residues in the RNA-binding region due to conformational sampling in the μs-ms range. Overall, the ensemble of models aligns well with our experimental data, providing a robust structural hypothesis for calcium-activated, RNA-bound EndoU.

## Discussion

**Figure 5:**
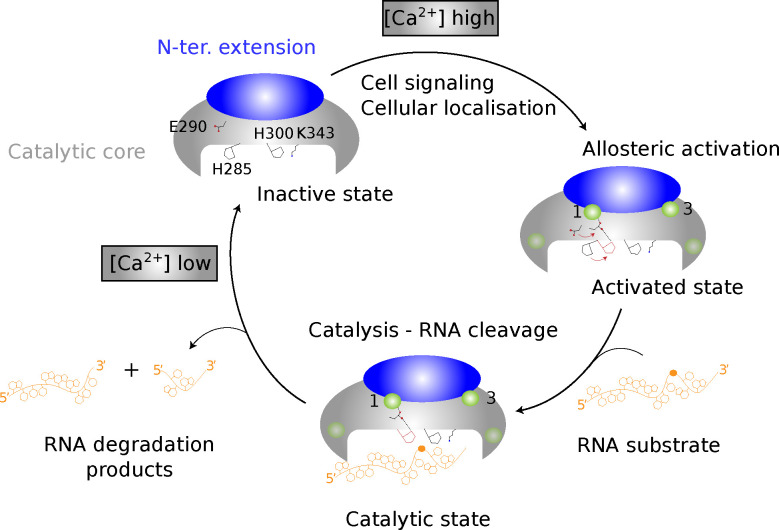
Schematic representation of eukaryotic EndoU activation upon calcium and substrate binding.

In this work, we elucidated the molecular basis for the Ca^2+^-dependent activation of human EndoU, with implications for the entire eukaryotic EndoU family due to the conserved sequence and structure of its catalytic domain across eukarya. Our findings, in conjunction with existing data, suggest an allosteric rather than catalytic requirement for a divalent metal in EndoU cleavage (Fig. 5).

Indeed, a common evolutionary origin with the RNase A family has been proposed based on structural and distant sequence similarities [6]. RNase A enzymes use a catalytic triad of two histidines and a lysine [68], and the *Xenopus* EndoU structure suggested a mechanistic similarity, where conserved His and Lys residues mark the proposed catalytic site [34]. Mutation of the corresponding residues in human EndoU supported these assignments (Fig. 3 C). Then, both EndoU and RNase A leaves a 5′-OH product, which is characteristic of metal-independent endonucleolytic catalysis [69]. Also, bacterial and some viral EndoU homologs do not require divalent metal ions [31, 11]. In the context of eukaryotic EndoU, our experimental data identified that calcium binding was necessary for cleavage activity in both mouse and human EndoU (Fig. 2, 3 C), with coordinating residues from both the conserved catalytic core and the eukaryote-specific N-terminal extension. We explained how the binding of calcium at the interface between the two regions could trigger water-mediated intramolecular signaling, ultimately leading to local structural changes that result in the positioning of catalytic residues in an active conformation. Therefore, our work supports a model where calcium is not directly involved in catalysis but rather activates the catalytic His-His-Lys triad through allostery. At the same time, we assign a role to the eukaryotic-specific N-terminal extension in sensing calcium at relevant sites, enabling allosteric regulation by calcium ions.

Eukaryotic EndoU proteins primarily depend on calcium for activation, as our data for mouse and human EndoU indicate exclusive activation by Ca^2+^, consistent with *Xenopus* EndoU findings [25]. Although early *Xenopus* studies also reported Mn^2+^ requirements, Ca^2+^-dependent cleavage was observed [13], and the *C. elegans* endu-2 homolog is activated by both Ca^2+^ and Mn^2+^ [70]. The physiological relevance of Mn^2+^ activation is unclear due to its low concentration in mammalian tissues [71, 72, 73], whereas Ca^2+^ concentrations vary significantly, making EndoU localization crucial for understanding Ca^2+^-mediated activation. Animal EndoUs contain an N-terminal signal peptide, and mammalian EndoUs have two somatomedin B (SmB) domains rich in disulfide bonds, suggesting secretion or ER association [74, 75, 76]. The *C. elegans* endu-2 and *D. melanogaster* CG2145 homologs are secreted and reuptaken in other tissues [70, 77], while *Xenopus* and human EndoU are cytoplasmic and ER-associated [25]. Calcium, a universal eukaryotic second messenger, is near zero in resting cells and rises to 1–100 μM concentration during signaling, while extracellular Ca^2+^ can reach mM levels [78]. EndoU may cleave RNAs or alter mRNA expression during thymocyte maturation or apoptosis, consistent with its proposed pro-apoptotic role in B cells [27]. The Ca^2+^ activation mechanism is likely conserved across eukaryotic EndoUs, with varying expression domains and RNA targets across species. If delivered to Ca^2+^-rich extracellular environments, EndoU may degrade extracellular RNAs, which makes the understanding of the RNA targeting repertoire of EndoU a priority to further illuminate its biological roles.

Our work provides fundamental insights with potential applications in therapeutic and biotechnological domains. The Nsp15 protein of SARS-CoV-2 features a nidovirus EndoU-like domain (NendoU) that requires divalent manganese ions to cleave the 5′-polyuridine tract of its negative-sense RNA, a crucial process for evading the host immune system [79]. Tipiracil, a uridine analog, effectively binds to the uridine binding site of Nsp15, inhibiting its activity and diminishing Spike protein expression in whole-cell assays, thereby inhibiting SARS-CoV-2 [80]. However, the structural basis for the divalent metal ion dependence of Nsp15 remains unknown, posing challenges for rational drug design targeting Nsp15 and other members of the EndoU-like superfamily regulated by divalent metal ions. By elucidating the structural basis of the divalent metal ion dependence of human EndoU, we pave the way for developing inhibitors specifically targeting EndoU-like domains that depend on these ions. Furthermore, investigating whether Tipiracil can bind to and inhibit human EndoU could provide valuable insights into the cellular and extracellular functions of EndoU, potentially identifying it as a pharmacological target in certain disease contexts. The potential of EndoU as a biotechnological tool for specific RNA sequence cleavage is noteworthy, especially with advancements in self-driving laboratories and machine learning algorithms for enzyme design and optimization [81, 82, 83]. Altering the divalent metal ion dependence of EndoU could yield sensors for detecting environmental contaminants like lead ions in water or enable controlled RNA digestion in biological systems by introducing activating ions, offering significant insights into RNA metabolism and processes.

In conclusion, we have elucidated the molecular mechanism underlying the activation of EndoU enzymes by divalent metal ions, specifically calcium. This discovery demonstrates a functional role for the N-terminal extension in human EndoU, suggesting a conserved mechanism accross eukaryotic EndoU catalytic domains. Evolution has provided these enzymes with a distinct regulatory segment that interacts with their catalytic core to sense calcium binding and communicate with the catalytic site through allostery. Our findings pave the way for more research into the role of EndoU in biological processes and offer valuable insights for future biotechnological and therapeutic applications.

## Data Availability

The atomic coordinates of the calcium-activated EndoU structure have been deposited in the PDB under the accession code 9FTW.
